# Protective Effect of Electroacupuncture on the Barrier Function of Intestinal Injury in Endotoxemia through HO-1/PINK1 Pathway-Mediated Mitochondrial Dynamics Regulation

**DOI:** 10.1155/2023/1464853

**Published:** 2023-01-07

**Authors:** Yuan Zhang, Zhenzhen Meng, Lina Wu, Xiuyun Liu, Chenxu Guo, Jianbo Yu, Dong Ming, Shuan Dong, Yingya Cao, Xianhong Jiang

**Affiliations:** ^1^Department of Anesthesiology and Critical Care Medicine, Tianjin Nankai Hospital, Tianjin Medical University, Tianjin 300100, China; ^2^Department of Biomedical Engineering, College of Precision Instruments and Optoelectronics Engineering, Tianjin University, Tianjin 300072, China; ^3^Academy of Medical Engineering and Translational Medicine, Tianjin University, Tianjin 300072, China

## Abstract

**Background and Aims:**

Endotoxemia (ET) is a common critical illness in patients receiving intensive care and is associated with high mortality and prolonged hospital stay. The intestinal epithelial cell dysfunction is regarded as the “engine” of deteriorated ET. Although electroacupuncture (EA) can mitigate endotoxin-induced intestinal epithelial cell dysfunction in ET, the mechanism through which EA improves endotoxin-induced intestinal injury for preventing ET deterioration needs further investigation.

**Methods:**

An in vivo ET model was developed by injecting lipopolysaccharide (LPS) in wild-type and PINK1-knockout mice. An in vitro model was also established by incubating epithelial cells in the serum samples obtained from both groups of mice. Hemin and zinc protoporphyrin IX (ZnPP) were applied to activate or inhibit heme oxygenase 1 (HO-1) production. EA treatment was performed for 30 min consecutively for 5 days before LPS injection, and on the day of the experiment, EA was performed throughout the process. Samples were harvested at 6 h after LPS induction for analyzing tissue injury, oxidative stress, ATP production, activity of diamine oxidase (DAO), and changes in the levels of HO-1, PTEN-induced putative kinase 1 (PINK1), mitochondrial fusion and fission marker gene, caspase-1, and interleukin 1 beta (IL-1*β*).

**Results:**

In the wild-type models (both in vivo and vitro), EA alleviated LPS-induced intestinal injury and mitochondrial dysfunction, as indicated by decreased reactive oxygen species (ROS) production and oxygen consumption rate (OCR) and reduced levels of mitochondrial fission proteins. EA treatment also boosted histopathological morphology, ATP levels, DAO activity, and levels of mitochondrial fusion proteins in vivo and vitro. The effect of EA was enhanced by hemin but suppressed by Znpp. However, EA + AP, Znpp, or hemin had no effects on the LPS-induced, PINK1-knocked out mouse models.

**Conclusion:**

EA may improve the HO-1/PINK1 pathway-mediated mitochondrial dynamic balance to protect the intestinal barrier in patients with ET.

## 1. Introduction

Endotoxemia (ET) is common in clinically ill patients receiving intensive care and can easily induce multiple organ dysfunction syndromes (MODS) and threaten patients' life. Intestinal epithelial cell dysfunction is considered the main cause of systemic inflammation, such as MODS after ET. Early and effective interventions to prevent gut barrier dysfunction have been proved to be beneficial for patients with ET [1].

The mitochondria are highly dynamic organelles that undergo delicate fusion and fission cycles to maintain their functions and shape when cells experience metabolic or environmental stress [2]. In mammals, mitochondrial fusion is regulated by mitofusin 1 and 2 (Mfn1/2), situated in the outer membranes of the mitochondria, and optic atrophy 1 (OpA-1), situated in the inner membranes of the mitochondria. Mitochondrial fission is promoted by dynamin-related protein 1 (Drp1) in the cytoplasm and mitochondrial division protein 1 (Fis1) located in the outer membrane of the mitochondria [2, 3]. Dysregulation of these processes results in an abnormally fused or fragmented mitochondrial network that is unable to meet cellular energy demands.

Dysregulated mitochondrial dynamics has been found to be related to disease development and progression. Intestinal disease causing intestinal dysfunction has been reported to be related to disrupted mitochondrial dynamics [4]. Our previous studies have demonstrated that the mitochondria with a proper dynamic balance of fusion/fission could resist endotoxin-induced acute lung injury [5–7]. Previous studies have also proved that the progression of organ failure during sepsis is partially due to mitochondrial dysfunction initiated by oxidative stress, along with a decreased mitochondrial respiratory control ratio [8, 9]. However, whether modulating the mitochondrial fusion/fission balance can alleviate the gut barrier dysfunction in ET is currently unknown. Exploring a potential antioxidant to preserve mitochondrial dynamics may be a vital determinant for protecting the mitochondria from oxidative damage in animal models of sepsis-induced organ failure.

Heme oxygenase-1 (HO-1) can regulate mitochondrial dynamic equilibrium and impede oxidative cellular injury by catalyzing the breakdown of heme to free iron, carbon monoxide, and biliverdin in mammalian cells [10–12]. PTEN-induced putative kinase 1 (PINK1), the only kinase located mainly in the mitochondrial intima, has been found to have a certain protective effect on the mitochondria during cellular stress, which is important in the HO-1 pathway. However, the basic mechanism remains to be investigated [13–15].

**Figure 1:**
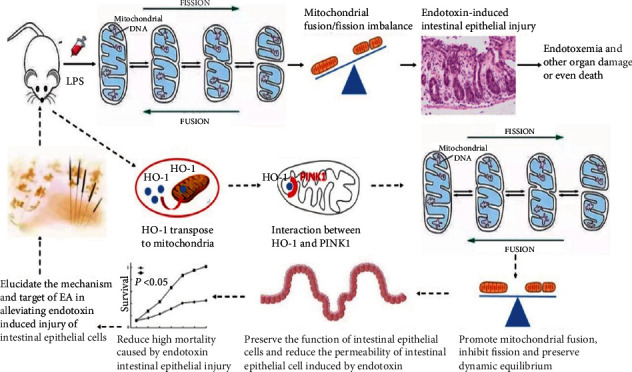
Outline of the experimental design.

In recent years, electroacupuncture (EA) on proper acupoints (AP) has gained popularity as a nonpharmaceutical treatment for intestinal injury [16–18]. Our previous studies have demonstrated the protective effect of EA on ET-induced lung injury, wherein we found an increase in HO-1 expression and mitochondrial fusion activities [19–21]. However, whether EA is effective in protecting intestinal epithelial cells in patients with ET by regulating the mitochondrial fusion/division homeostasis is currently unknown. Therefore, in the present study, we hypothesized that EA may protect the intestinal epithelial cells in patients with ET by regulating the mitochondrial fusion/fission dynamics and protect the intestinal barrier by activating the HO-1/PINK1 pathway. Both wild-type and PINK1-knockout mice and cells were used in the study, and an ET model was established both in vivo and vitro. We attempted to validate our hypothesis to explore the basic mechanism of EA treatment for ET (Figure 1).

## 2. Materials and Methods

### 2.1. Ethics Approval

All animal procedures were approved by the Animal Ethics and Welfare Committee of Nankai University (No.81803899) and performed according to the Guidelines for the Care and Use Experimental Animals.

### 2.2. Establishment of an In Vitro LPS-Induced Cell Intestinal Barrier Model

Human colon adenocarcinoma cells (Caco-2 cells) were cultured in the DMEM complete medium (containing 20% fetal bovine serum) at 37°C in a 5% CO_2_ incubator and passaged by trypsin digestion every 3–5 days. The cells were counted using a blood cell counting plate, which were seeded on the top of a Transwell chamber at a density of 2 × 10^5^ cells/ml. Then, 400 *μ*l of cells and 600 *μ*l of DMEM complete medium were added to each well. After inoculation, the medium was changed every day. The growth status of cells was observed by determining trans-epithelial electrical resistance (TEER). After 21 days of culture, the cells were dense and well-connected per our observation under a light microscope, and TEER was significantly increased and stabilized at 450–500 *Ω*/cm, which indicated that an in vitro Caco-2 cell intestinal barrier model was established successfully. The intestinal barrier model cells were further exposed to LPS (100 *μ*g/mL, dissolved in 1 mL of 0.9% normal saline) for 24 h to establish an LPS-induced cell intestinal barrier model in vitro.

A certain amount of blood was taken from the heart of the mice treated with EA on AP via a 5 mL vacuum blood sampling needle. The blood was cooled for 1 h and centrifuged (2,500 rpm) for 25 min. The sediment was discarded, and the serum was retained. The serum was inactivated in a water bath at 56°C for 30 min and then sterilized using 0.22 *μ*M semipermeable membranes. The prepared serum was added to the Caco-2 cell intestinal barrier model at a ratio of 10%. After 12 h of incubation, the Caco-2 cell intestinal barrier model with EA on AP intervention was established [22]. The Caco-2 cells were subcultured for three passages. Tests were performed at each passage. Each test was repeated three times. Mycoplasma detection was performed through PCR analysis to ensure that the cells were not infected by mycoplasma [23].

### 2.3. Establishment of the Mouse Intestinal Injury Model of ET

Eight-week-old, male C57BL/6 mice (weighing 18 to 22 g) were provided by the Laboratory Animal Center of Nankai University, Tianjin, China. The mice were caged individually at 30%–70% humidity (18 to 25°C) and acclimatized to a 12 h light-dark cycle, with access to food and water ad libitum. The animals were anesthetized and operated, as described previously [24]. To induce intestinal injury, 10 mg/kg LPS (dissolved in 1 mL of 0.9% normal saline) was injected intraperitoneally, and the control group was injected with an equal volume of normal saline. Further experimental details were described in our previous study [23]. The mice were sacrificed 6 h after the LPS injection, and the specimens were isolated for further analysis (the animals who died within 6 h were excluded). The mice were acclimated for three days prior to the experiments.

### 2.4. Different Groups of Cells and Mice with/without Treatment

The cells were divided into 5 groups, namely control, LPS (LPS-induced cells), LPS + EA + AP group (LPS cells treated with EA on AP targets), LPS + EA + AP + ZnPP group (LPS cells treated with EA on AP targets and incubated with ZnPP), and LPS + EA + AP + hemin group (LPS cells treated with EA on AP targets and incubated with hemin). For EA intervention, the cells were cultured with the serum obtained from the LPS mice treated with EA. The cells were digested after 6 h. Then, the cell pellets were resuspended in 1 mL phosphate buffer saline for complete homogenization. The supernatants of the cell homogenate were extracted by centrifugation at 4°C and 15,000 rpm for 10 min. The samples were frozen in liquid nitrogen and stored at -80°C until further assays.

The mice were divided into 5 groups, with 10 mice in each group: Control group (wild mice injected with normal saline), LPS group (mice injected with LPS), LPS + EA + AP group (LPS mice treated with EA on AP targets), LPS + EA + AP + ZnPP group (LPS mice treated with EA on AP targets and injected with ZnPP), and LPS + EA + AP + hemin group (LPS mice treated with EA on AP targets and injected with hemin). For the LPS + AP + EA group, the bilateral acupoints of Zusanli at 5 mm depth and Hegu at 1 mm depth were selected as the AP locations (for more details, please refer to supplementary materials and methods). During the treatment period, the needle handles were connected to a Han's nerve AP stimulator with a disperse-dense wave of 2 Hz/15 Hz frequency and 1 mA intensity applied. A daily 30 min EA treatment was performed for 5 days before administering the LPS injection. On the day of the experiment, EA was performed 30 min before the LPS injection. The needles were retained in the APs until the end of the experiment. On the other hand, the LPS + EA group received EA treatment on locations beyond the selected targets. An experienced acupuncturist identified the AP and non-AP targets. In the groups treated with drugs, ZnPP or hemin was injected via the tail vein; hemin was injected at the dose of 100 mg/kg dissolved in 1 mL of NaOH, and Znpp was injected at a dose of 10 *μ*mol/kg dissolved in 1 mL of NaHCO_3_.

All the mice were euthanized 6 h after LPS injection, and a 10 cm long small intestine was dissected from the terminal ileum. The small intestine was carved along with the antimesenteric attachment, and a mucosal homogenate was obtained. The mucosal homogenate (1 mL) was centrifuged at 15,000 rpm and 4°C for 5 min, and the cell pellets were resuspended in 1 mL phosphate buffer saline for complete homogenization. Then, the supernatant of the cell homogenate was extracted by centrifugation at 4°C and 15,000 rpm for 10 min. The samples were frozen in liquid nitrogen and stored at -80°C until further analysis.

### 2.5. Determining Mitochondrial Function

The level of reactive oxygen species (ROS) in the intestinal mucosal epithelial cells was determined spectrofluorometrically using the DCFH-DA as a fluorescent dye. Briefly, the intestine mucosal cell suspension was treated with 10 *μ*mol/L DCFH-DA at 37°C for 30 min in the dark. DCF fluorescence was monitored with Ex/Emof 480 nm/530 nm by using the Chameleon microplate reader (Hidex, Turku, Finland). The fluorescence intensity of the sample was determined as the differences relative to the initial fluorescence. The oxygen consumption rate (OCR) of the intestinal mucosal epithelial cells was detected using the Cayman oxygen consumption rate detection kit and analyzed by flow cytometer (Beckman Coulter, CA, USA). The data were analyzed using FlowJo software (Tree Star, OR, USA). The ATP level was determined using an ATP assay kit, and the absorbance of every well was monitored at the optical density (OD) of 570 nm by using a microplate reader (Bio-Tek Instruments, VT, USA) [25].

### 2.6. Assessment of the Extent of Intestinal Epithelial Cell Injury

The extent of intestinal epithelial cell injury was assessed by determining the diamine oxidase (DAO) levels. The activity of DAO was determined by ELISA. The activity of DAO in animal plasma and cell culture supernatant samples was determined using the double antibody sandwich method. The specific methods used are as follows: first, the microplates were coated using the purified DAO antibody to make a solid-phase antibody. Second, standard DAO and animal plasma and cell culture supernatants were added to the coated microplates. Third, HRP-labeled DAO antibody was added to the microplates to form antibody antigen enzyme-labeled antibody complexes. After thorough washing, the substrate 3,3′,5,5′ -tetramethylbenzidine (TMB) of HR was added for color development. The color of TMB turned blue because of the catalysis of the HRP enzyme and turned yellow under the action of the acid. The color density was positively correlated with the activity of DAO. The absorbance (OD value) was measured at a wavelength of 450 nm by using a microplate reader. The activity of DAO was calculated by determining the standard curve.

### 2.7. Real-Time Quantitative Reverse Transcription PCR

Total RNA was isolated from cultured Caco-2 cells or mouse intestine mucosa by using a high-purity RNA kit (Roche, Switzerland). Total RNA was quantified using a spectrophotometer at 260 nm. Then, 2 *μ*g of total RNA was reverse transcribed to produce first-strand cDNA by using the SYBR Green Master Mix on the ABI Prism 7000 sequence detector system (Applied Biosystems, Foster City, USA). The PCR conditions were as follows: predegeneration at 95°C for 10 min, followed by 40 thermal cycles of denaturation for 30 s at 95°C; annealing for 5 s at 95°C; and extension for 30 s at 60°C. Specific primers used for marker genes of the status of the mitochondrial dynamic equilibrium are described in Supplementary Methods. The housekeeping gene *β*-actin served as an internal control to normalize all PCR products. Target gene expression was quantified using the comparative cycle threshold (C_T_) methods [26, 27].

### 2.8. Western Blot Analysis

At the indicated time after pretreatment with various factors, the cultured Caco-2 cells or mouse intestine mucosa were homogenized in the ice-cold lysis buffer for 30 min. The protein extracts were centrifuged at 4°C and 15,000 rpm for 15 min, and the supernatants were quantified using the bicinchoninic acid protein assay kit (Thermo Fisher Scientific, MA, USA). Equal amounts of protein (50 *μ*g per lane) were separated by 12% SDS-PAGE and then transferred to PVDF membranes (Bio-Rad, Hercules, CA, USA) by using the Bio-Rad transblot apparatus. The blots were washed with 1X Tris-buffered saline (TBS; 200 mmol/L Tris and 1.5 mol/L NaCl) three times and then blocked using 5% skim milk for 40 min at room temperature. The proteins were incubated overnight at 4°C with primary antibodies against HO-1 (1 : 500), PINK-1 (1 : 500), Drp1 (1 : 500), Mfn1 (1 : 400), Mfn2 (1 : 300), Fis1 (1 : 500), OPA1 (1 : 500), caspase-1 (1 : 500), IL-1*β* (1 : 500), and *β*-actin (1 : 500). The blots were washed with TBS-0.05% Tween 20 three times and incubated for 2 h at 37°C with HRP-conjugated secondary antibody (1 : 300). The protein signal was visualized using the Immobilon Western Chemiluminescent HRP Substrate detection reagent (Millipore, MA, USA) and imaged using Image Lab software (Bio-Rad, VA, USA). Finally, the proteins were quantified through densitometry using the Molecular Analyst Image-analysis Software (Bio-Rad Laboratories, CA, USA). The protein concentration of the marker genes was normalized to *β*-actin concentration by using the OD ratio.

### 2.9. Histopathological Examination of Cells and Observation of Mitochondrial Ultrastructure

After sacrificing the mice, 2 jejunum tissues were extracted from each mouse, of which one was used for histopathological examination and the other for observing the change in the mitochondrial ultrastructure. For the tissue histopathological examination, the jejunum tissues were cut into 5 mm sections, sliced into thin slices, fixed in 10% formalin, embedded in paraffin, and stained with hematoxylin and eosin (H&E) dye. We then evaluated the extent of injury to the intestine mucosa tissue according to inflammatory cell infiltration. To observe changes in the mitochondrial ultrastructure, the mucosal layer of the injured jejunum was fixed in 2.5% glutaraldehyde overnight at 4°C and then fixed in 1% aqueous osmium for 1 h, dehydrated with acetone, and embedded in resin based on routine protocols. The ultra-thin sections (<100 nm) were prepared using a microtome and mounted on copper grids. The mitochondrial ultrastructure was observed under a transmission electron microscope.

## 3. Results

### 3.1. The Mechanism of EA + AP Treatment in LPS-Induced Intestinal Injury In Vivo and In Vitro

**Figure 2:**
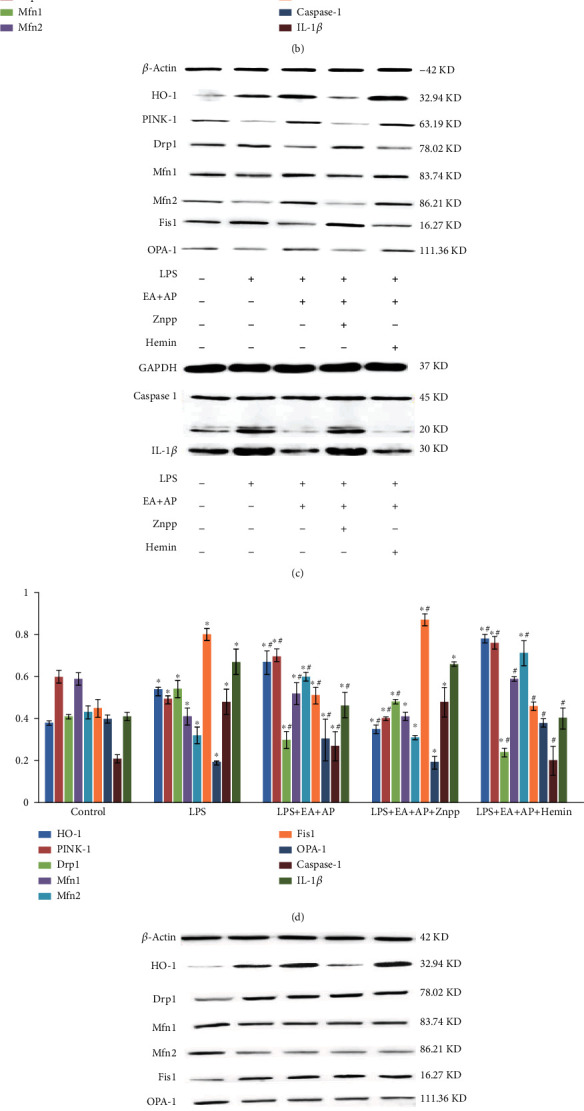
The levels of mRNAs (a, b) and proteins (c, d, e, and f) known as mitochondrial fusion/fission markers and pyroptosis factors in the wild-type mice (a, c, and d) and PINK1-knockout mice (b, e, and f). HO-1: heme oxygenase-1, PINK1: PTEN-induced putative kinase 1, Drp1: the dynamin-related protein 1, Mfn1/2: the mitofusin 1 and 2, Fis1: mitochondrial division protein 1, OpA-1: the optic atrophy 1, IL-1*β*: interleukin-1*β*, LPS: lipopolysaccharide, EA + AP: electroacupuncture (EA) performed on acupoint (AP), hemin: a substrate and inducer of HO-1, and ZnPP: zinc protoporphyrin IX, an inhibitor of HO-1, +/-. The mice were or were not treated with corresponding factors. *β*-Actin served as an internal standard to ensure similar gel loading of the starting material in each sample. The mRNA and protein levels are compared using the paired sample *t*-test. ^∗^: significant difference compared with the control mice (*P* < 0.05). ^#^: significant difference compared with the LPS-exposed mice (*P* < 0.05).

**Figure 3:**
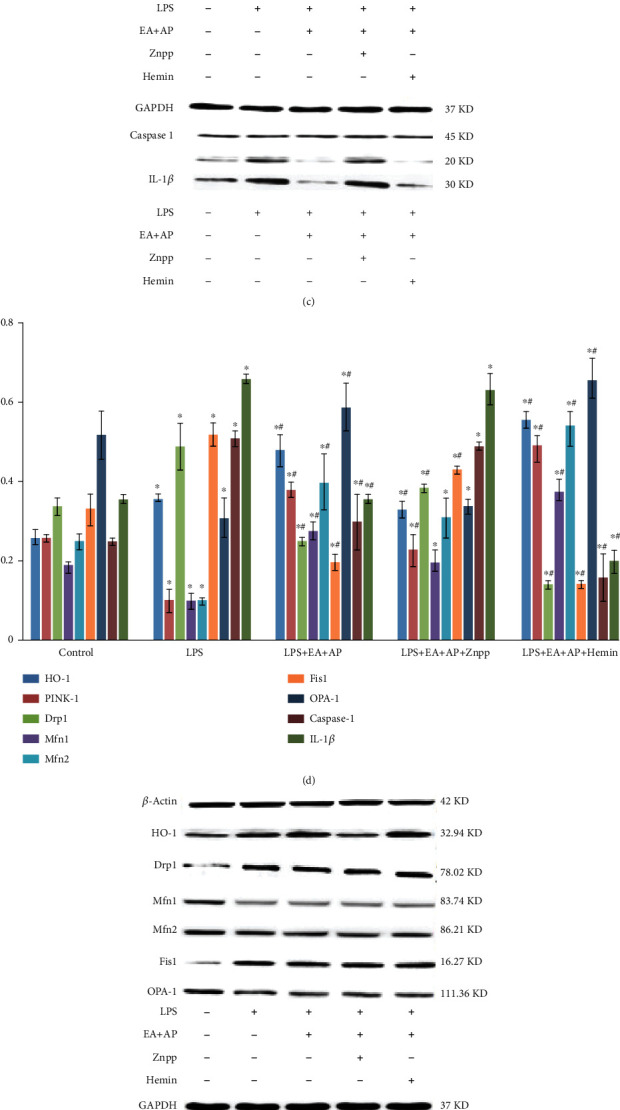
The levels of mRNAs (a, b) and proteins (c, d, e, and f) known as mitochondrial fusion/fission markers and pyroptosis factors in the wild-type Caco-2 cells (a, c, and d) and PINK1-knockout Caco-2 cells (b, e, and f). HO-1: heme oxygenase-1, PINK1: PTEN-induced putative kinase 1, Drp1: the dynamin-related protein 1, Mfn1/2: the mitofusin 1 and 2, Fis1: mitochondrial division protein 1, OpA-1: the optic atrophy 1, IL-1*β*: interleukin-1*β*, LPS: lipopolysaccharide, EA + AP: electroacupuncture (EA) performed on acupoint (AP), hemin: a substrate and inducer of HO-1, and ZnPP: zinc protoporphyrin IX, an inhibitor of HO-1, +/-. The cells were or were not treated with corresponding factors. *β*-Actin served as an internal standard to ensure similar gel loading of the starting material in each sample. The mRNA and protein levels were compared using the paired sample *t*-test. ^∗^: significant difference compared with the control cells (*P* < 0.05). ^#^: significant difference compared with the LPS-exposed cells (*P* < 0.05).

The LPS-treated wild-type mice or cells and PINK1-knockout mice or cells were used to understand the mechanism of EA + AP treatment in LPS-induced intestinal injury both *in vivo* and *in vitro* (Figures 2 and 3). Figures 2(a), 2(c), and 2(d) and Figures 3(a), 3(c), and 3(d) show that LPS injection downregulated HO-1, PINK1, Mfn1, Mfn2, and OPA-1 mRNA expressions, decreasing respective protein levels (*P* < 0.05), and upregulated Drp1, Fis1, caspase-1, and IL-1*β* mRNA expressions, increasing respective protein levels, in the wild-type mice or cells (*P* < 0.05). When the wild-type mice and cells were subjected to EA + AP treatment, the mRNA and protein levels induced by LPS were alleviated (Figures 2 and 3; *P* < 0.05). Znpp (the HO-1 inhibitor) and hemin (a substrate and potent inducer of HO-1) were used alone to treat the wild-type mice and cells. The effect of EA + AP was reversed by the HO-1 inhibitor Znpp in the wild-type mice and cells; however, the HO-1 substrate and potent inducer hemin reversed the inhibitory effect of Znpp (Figures 2 and 3; *P* < 0.05).

However, the EA + AP, Znpp, and hemin treatments did not affect Drp1, Mfn1, Mfn2, Fis1, OPA-1, caspase-1, and IL-1*β* mRNA expressions and respective protein levels in the LPS-induced and PINK1-knockout mice and cells. (Figures 2(b), 2(e), and 2(f) and Figures 3(b), 3(e), and 3(f)).

### 3.2. EA Exerted a Protective Effect by Regulating the HO-1/PINK1 Pathway in LPS-Induced Mitochondrial Dysfunction and Intestinal Epithelial Cell Injury In Vivo and In Vitro

**Figure 4:**
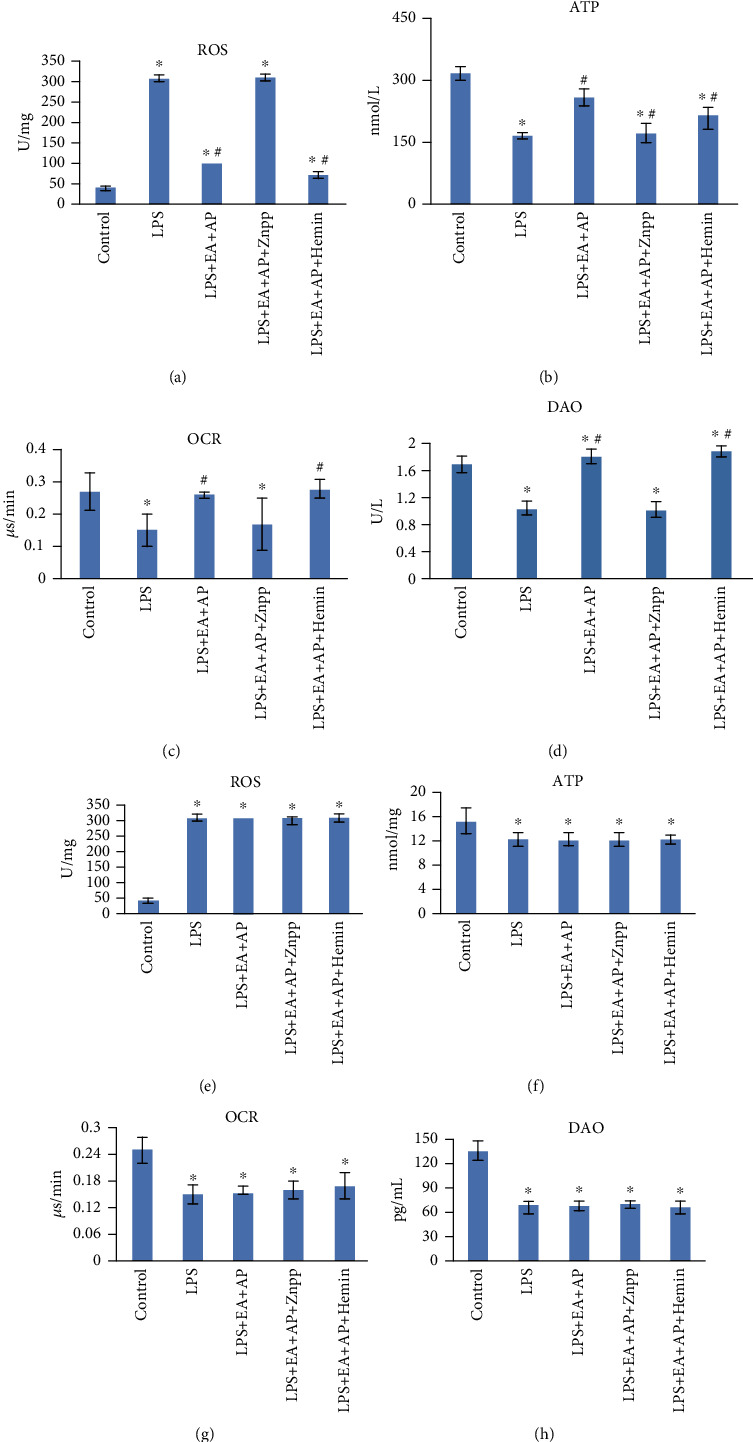
Effect of EA on ROS production, ATP content, OCR, and DAO levels in the LPS-exposed wild-type (a, b, c, and d) and PINK1-knockout mice (e, f, g, and h). LPS: lipopolysaccharide, EA + AP: electroacupuncture (EA) on acupoint (AP), hemin: a substrate and inducer of HO-1, ZnPP: zinc protoporphyrin IX, an inhibitor of HO-1, OCR: oxygen consumption rate, ROS: reactive oxygen species, DAO: diamine oxidase, and ATP: adenosine triphosphate. The mRNA and protein levels were compared using the paired sample *t*-test. ^∗^: significant difference compared with the control mice (*P* < 0.05). ^#^: significant difference compared with the LPS-exposed mice (*P* < 0.05).

**Figure 5:**
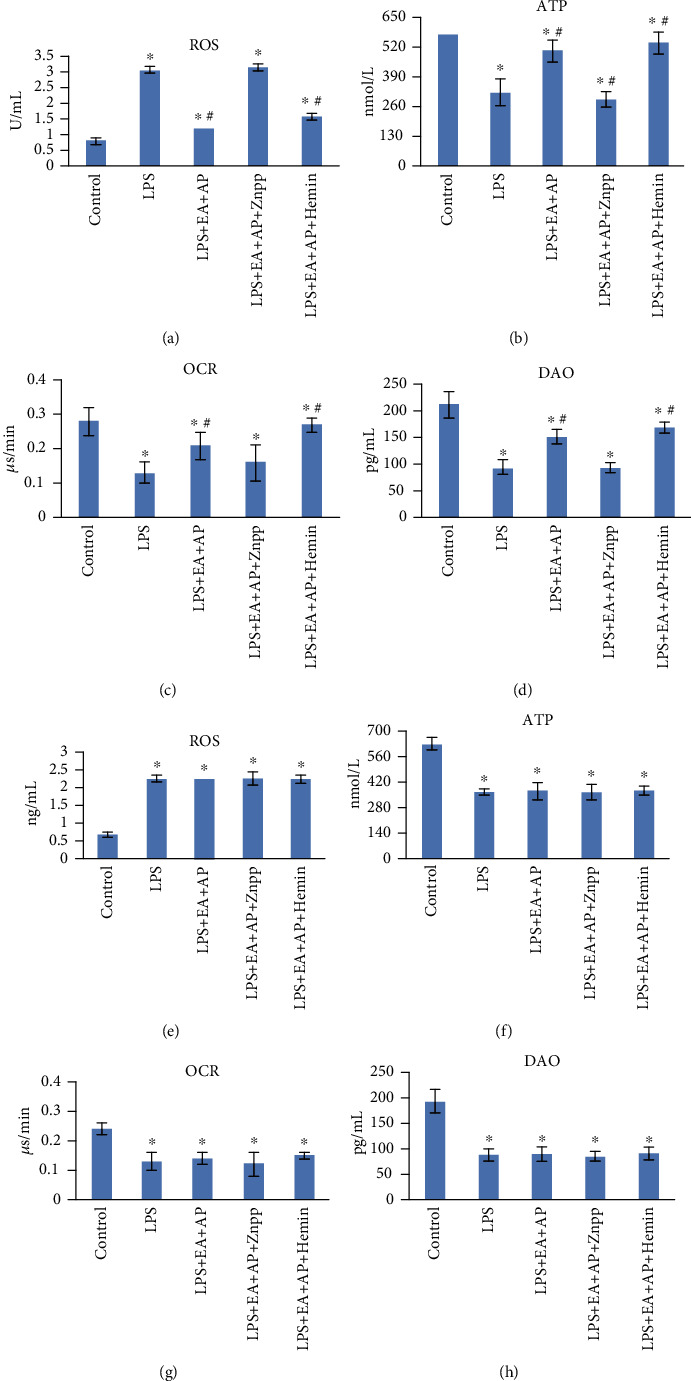
Effect of EA on ROS production, ATP content, OCR, and DAO levels in the LPS-exposed wild-type (a, b, c, and d) and PINK1-knockout mice (e, f, g, and h). LPS: lipopolysaccharide, EA + AP: electroacupuncture (EA) on acupoint (AP), hemin: a substrate and inducer of HO-1, ZnPP: zinc protoporphyrin IX, an inhibitor of HO-1, OCR: oxygen consumption rate, ROS: reactive oxygen species, DAO: diamine oxidase, and ATP: adenosine triphosphate. The mRNA and protein levels were compared using the paired sample *t*-test. ^∗^: significant difference compared with the control cells (*P* < 0.05). ^#^: significant difference compared with the LPS-exposed cells (*P* < 0.05).

The four indices of mitochondrial function and intestinal epithelial cell injury degree, namely OCR, ROS, ATP, and DAO, were compared between the wild-type and PINK1-knockout mice and cells. As shown in Figures 4 and 5, LPS increased the ROS contents in the mitochondria (Figures 4(a), 4(e), 5(a), 5(e)), whereas it decreased the ATP (Figures 4(b), 4(f), 5(b), 5(f)), OCR (Figures 4(c), 4(g), 5(c), 5(g)), and DAO (Figures 4(d), 4(h), 5(d), 5(h)) levels (*P* < 0.05) in both the wild-type and PINK1-knockout mice and cells. The EA effect on the wild-type cells was reversed by Znpp, whereas hemin reinforced the effect of EA + AP in the wild-type mice and cells. EA + AP, Znpp, and hemin did not affect the LPS-induced and PINK1-knockout mice and cells.

### 3.3. EA Changed the Histopathological Morphology of LPS-Induced Intestinal Epithelium Tissues in the Wild-Type Mice

**Figure 6:**
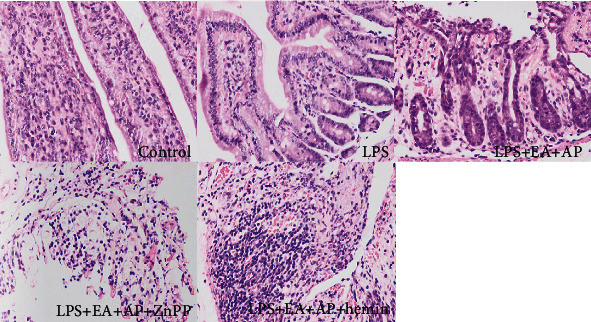
Microphotographs of histopathologic changes in the small intestine epithelial tissue stained with H&E. Representative images of H&E-stained small intestinal epithelial slices from the control group (mice were injected with normal saline instead of LPS), LPS group (the mouse intestinal injury model of ET), LPS + EA + AP group (the mouse intestinal injury model of ET treated by EA on AP), LPS + EA + AP + ZnPP group (the LPS +EA + AP group was injected with ZnPP), and LPS + EA + AP + hemin group (the LPS +EA + AP group was injected with hemin). H&E: hematoxylin and eosindye, LPS: lipopolysaccharide, EA: electroacupuncture, AP: acupoint, ET: endotoxemia, and Znpp: zinc protoporphyrin IX.

As shown in Figure 6, the LPS group showed a decreased number of normal cells in the jejunal epithelial tissue than the control group. The tissues in the LPS group showed cellular vacuolization, swelling, desquamation, and interstitial edema. In the LPS + EA + AP group, the degree of the jejunal epithelial tissue injury was attenuated to a certain extent, which was shown by the increased normal cell density and reduced cellular vacuolization, swelling, desquamation, and interstitial edema. Additional treatment with hemin augmented the effects of EA + AP. However, additional treatment with ZnPP weakened the protective effects of EA + AP (the LPS + EA + AP + ZnPP group), and hemin reinforced the effect of EA + AP against the LPS-induced jejunal epithelial tissue injury.

### 3.4. EA Changed the Mitochondrial Ultrastructure in the Wild-Type Mice

**Figure 7:**
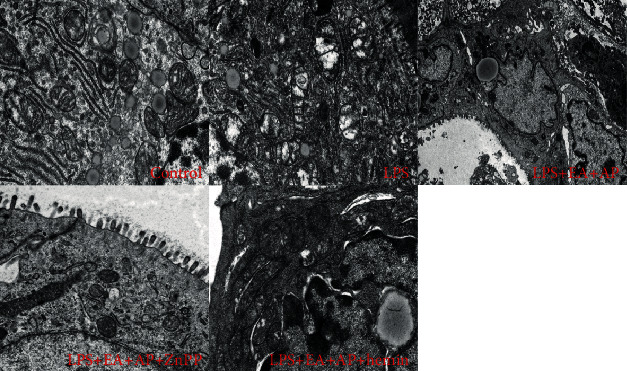
The change in the ultrastructure of the mitochondria in the small intestine epithelial cells. Electron microscopy images of the mitochondria in small intestine epithelial tissues from the control group (the mice were injected with normal saline instead of LPS), LPS group (the mouse intestinal injury model of ET), LPS + EA + AP group (the mouse intestinal injury model of ET treated by EA on AP), LPS + EA + AP + ZnPP group (the LPS + EA + AP group was injected with ZnPP), and LPS + EA + AP + hemin group (the LPS+ EA + AP group was injected with hemin). LPS: lipopolysaccharide, EA: electroacupuncture, AP: acupoint, ET: endotoxemia, and Znpp: zinc protoporphyrin IX.

As shown in Figure 7, LPS-induced mitochondrial edema and crest fracture (the LPS group) were attenuated by EA + AP treatment (the LPS+ EA + AP group) and further attenuated by hemin (the LPS + EA + AP + hemin group). However, Znpp partially inhibited the protective effects of EA + AP (the LPS + EA + AP + Znpp group).

## 4. Discussion

This study showed that EA exerted a protective effect on ET-induced intestinal injury by regulating the HO-1/PINK1 pathway-mediated mitochondrial dynamics both *in vitro* and *in vivo*. The study showed that EA + AP can regulate the mitochondrial fusion/fission balance and preserve the mitochondrial function by increasing ATP production, DAO activity, and OCR; upregulating mitochondrial fusion marker gene expression; increasing respective protein levels; decreasing the ROS content and OCR; and downregulating mitochondrial fission marker gene expressions and the respective protein levels. The study also showed that the HO-1 inhibitor mitigates the positive effect of EA + AP on LPS-induced intestinal injury and that the positive effect of EA + AP can be enhanced by the HO-1 inducer hemin. To further investigate the role of HO-1, we used PINK1-knockout models and found that the protective effect of EA + AP against endotoxin-induced intestinal injury disappeared. The function of the intestinal epithelial barrier mainly depends on the balance between apoptosis and proliferation of intestinal epithelial cells [28]. Usually, the dynamic renewal of intestinal epithelial cells plays an important role in maintaining the normal structure and function of the intestinal epithelium. The destruction of the inherent dynamic balance of intestinal epithelial cells can cause intestinal injury. ET induces inflammatory factors in the gastrointestinal microenvironment, which can facilitate apoptosis by activating the proapoptotic signal in intestinal epithelial cells via the cell membrane death receptor [29]. Excessive apoptosis of intestinal epithelial cells may disrupt the intestinal epithelial barrier function and accelerate the migration of intestinal bacteria from the intestine to the systemic blood circulation along the mesenteric lymphatic vessels, thus inducing ET deterioration and MODS development [30].

Pyroptosis is a form of programmed cell death characterized by the cysteinyl aspartate-specific proteases-1 (caspase-1) and the release of a large number of proinflammatory cytokines such as IL-1*β* [31–33]. Pyroptosis plays a role in the occurrence and development of infectious diseases including ET. The mitochondrion is the central link in the regulatory mechanism of apoptosis, in which tube network fragmentation occurs in the early stage of apoptosis [34]. Studies have proposed the concept of “microcirculation and mitochondrial distress syndrome”, pointing out the fundamental role of mitochondrial dysfunction in ET pathogenesis [35].

The stability of mitochondrial dynamics (the dynamic process of mitochondrial fusion and division) is important to ensure the normal spatial structure and morphology of the mitochondria [36]. In cells, mitochondria form a dynamic network structure by connecting with each other, which may represent an efficient energy-transport system or calcium channels between different regions of a cell. The dynamic balance of fusion/division maintains the stability of the morphological functions of mitochondria. Studies have shown that the imbalance of mitochondrial dynamics is involved in the occurrence and development of various acute/chronic major diseases. However, the underlying mechanism of mitochondrial dynamic destruction is unknown [37, 38]. Using the LPS-induced ET model, we showed that the mitochondrial fusion/division movement can be adjusted by regulating mitochondrial movement-related proteins, thus treating ET-induced intestine injury via restoring the mitochondrial dynamic balance [5, 7, 24].

ET increases free oxygen and nitrogen radicals and produces peroxynitrite. The persistently high levels of ROS and reactive nitrogen substances are harmful to the mitochondria, as they inhibit the mitochondrial respiratory chain function, thus reducing mitochondrial DNA replication and accelerating free radical generation. Hence, in this study, a vicious cycle of “free radical generation-mitochondrial structure destruction-free radical generation” was initiated. In the early stage of stress, fusion compensates for defects in mitochondria to maintain the energy needs. When mitochondrial damage exceeds a certain threshold, a mitochondrial division is much faster than fusion, resulting in the accumulation of many fragmented mitochondria in cells, which cannot be removed, causing irreversible damage to cells including intestinal epithelial cells [39, 40]. Therefore, the regulation of mitochondrial fusion/division and the maintenance of its dynamic stability are the prerequisite for the protection of the intestinal epithelial barrier function in ET.

Recent studies have shown that PINK1 plays an important role in regulating mitochondrial dynamics and mediating the autophagic clearance of damaged mitochondria [41, 42]. In this study, the positive effect of EA + AP was not observed on the ET-induced intestinal injury in the PINK1-knockout cells and mice. A possible reason is that HO-1 regulates mitochondrial homeostasis via PINK1 located in the mitochondrial intima.

## 5. Conclusions

EA + AP protects the intestine against endotoxin-induced injury by inducing HO-1 translocation to the mitochondrial intima, which may further regulate the mitochondrial fusion/division balance and protect the intestinal barrier function by activating PINK1. This study provides atheoretical basis for the novel therapy using EA on specific acupoints to treat ET-induced intestinal injury.

## Data Availability

Data is available on reasonable request though email (Dr. JB Yu, 30717008@nankai.edu.cn).

## Abbreviations

ET: Endotoxemia
MODS: Multiple organ dysfunction syndrome
HO-1: Heme oxygenase-1
ELISA: Enzyme-linked immunosorbent assay;
PTEN: Phosphatase and tensin homolog
PINK1: PTEN induced putative kinase 1
LPS: Lipopolysaccharide
EA: Electroacupuncture
AP: Acupoint
Fis1: One of the key mitochondrial fission protein members
Znpp: Zinc protoporphyrin IX
OCR: Oxygen consumption rate
ROS: Reactive oxygen species
DAO: Diamine oxidase
Mfn 1/2: Mitofusin 1/2
OPA1: Optic atrophy 1
Drp1: Dynamin-related protein 1.

## Supplementary Materials

Supplementary Materials Supplementary Table 1: primers of marker genes for qRT-PCR. Supplementary Figure 1: PINK1 gene knockout strategy. Supplementary Table 2: primers used for validating the efficiency of the designed cas9/sgRNA. Supplementary Table 3: the designed sgRNA sequences. Supplementary Figure 2: conversion products of EGE-WL-008-sgRNA4 and EGE-WL-008-sgRNA9.
