# Supplementary material for: Protective Effect of Electroacupuncture on the Barrier Function of Intestinal Injury in Endotoxemia through HO-1/PINK1 Pathway-Mediated Mitochondrial Dynamics Regulation
Source: Oxid Med Cell Longev. 2023 Jan 7;2023:1464853. doi: 10.1155/2023/1464853 (PMC9840552; doi:10.1155/2023/1464853)
Supplement: Supplementary Materials — Supplementary Table 1: primers of marker genes for qRT-PCR. Supplementary Figure 1: PINK1 gene knockout strategy. Supplementary Table 2: primers used for validating the efficiency of the designed cas9/sgRNA. Supplementary Table 3: the designed sgRNA sequences. Supplementary Figure 2: conversion products of EGE-WL-008-sgRNA4 and EGE-WL-008-sgRNA9. [file 1464853.f1.pdf]

Supplementary materials and methods

Reagents

Antibodies against HO-1 (s-10789), Mfn1 (sc-50330), Mfn2 (sc-50331), OPA-1 (sc-367890), Drp1 (sc32898), PINK1 (sc-518052), Fis1 (sc-376469), caspase-1 (sc-392736), IL-1β (sc-515598) and β-actin (sc-8432) were supplied by Santa Cruz Biotechnology (USA). The ROS-sensitive fluorescent dye 2′,7′-dichlorofluorescein diacetate (DCFH-DA) and ATP content test kits were obtained from Nanjing Jiancheng Bioengineering Institute (Nanjing, China). The diamine oxidase (DAO) activity detection kit was also bought from Nanjing Jiancheng Bioengineering Institute. The mitochondrial reductase function test, 3-(4,5-dimethylthiazol-2-yl)-2,5-diphenyltetrazolium bromide (MTT) assay kit was from Roche Applied Science (USA). The standards of lipopolysaccharide (LPS) (L1245), Znpp (a specific inhibitor of HO-1) (MKBV8659V) and hemin (BCBM3691V) were purchased from Sigma-Aldrich (St. Louis, MO, USA). Other chemical reagents were analytical grade.

Selection of the acupoints

Intestinal epithelial dysfunction caused by ET belongs to the categories of "stomach pain" and "fullness" in traditional Chinese medicine. Acupuncture and moxibustion therapy can invigorate the spleen and stomach, promote Qi and blood circulation, invigorate and strengthen the body, remove heat and detoxify, and exert its protective effect on the intestinal epithelial barrier in ET. The acupuncture points are mainly

selected from the Yangming meridian, such as Zusanli AP and Hegu AP [25, 27, 56]. The Zusanli AP matching with the Hegu AP has the effects of invigorating the spleen and stomach, promoting Qi and blood circulation, strengthening the body, reducing heat and detoxification. According to the "Animal Acupuncture Points Atlas" formulated by the Experimental Acupuncture Research Association of the Chinese Acupuncture and Moxibustion Society as well as mouse anatomy and body surface signs and our previous research, Zusanli AP of mouse is located at the lower lateral side of the knee joint, and 5mm below the head of fibula. Hegu AP of the mouse is located in the depression between the first and second metacarpal bones of forelimb. The needles are "Huatuo Brand" fine needles and are manufactured by Suzhou Medical Supplies Factory. The needles are with a length of 1.5 inches and a diameter of 0.30mm. The EA instrument is Han's nerve AP stimulator (HANS-200E, Nanjing Jisheng Medical Technology Co., Ltd., product standard No. YZB/Su-2008).

Primers of Real-time quantitative reverse transcription PCR

Specific primers used for marker genes of the status of the mitochondrial dynamic equilibrium were shown in Table 1. Housekeeping gene β-actin served as an internal control to normalize all PCR products.

Table 1. Primers of marker genes for qRT-PCR

Gene	Primer	Sequence（5'-3'）
HO-1	Forward	AGAGGAGATAGAGCGCAACAAG
	Reverse	CGTGGAGACGCTTTACATAGTG
PINK-1	Forward	GCACACTGTTCCTCGTTATGAA
	Reverse	AGGATGTTGTCGGACTTGAGAT
OPA-1	Forward	GAGAGTATCAAGCGGCACAAG

	Reverse	CCGATTCTTCCAGTACATCCA
Mfn1	Forward	ATAGAAGATGGCATGGGAAGAA
	Reverse	GTCCTCTTGAAAATCCGAACAC
Mfn2	Forward	CCAACATCTTCATCCTGAACAA
	Reverse	ACTTGAAAACCTTCTGCGAGAG
Fis1	Forward	TAAAGTATGTGCGAGGGCTGT
	Reverse	CAGGATTTGGACTTGGAGACA
Drp1	Forward	GACCCACTAGGTGGCCTTAAC
	Reverse	CCGTAACAATTCCTGTGTGCT
caspase-1	Forward	AGGATTTCTTAACGGATTC
	Reverse	ATAAAATCTTTCTCTATATGGGGC
IL-1β	Forward	AGGATCTCCACTACAGGCTGCG
	Reverse	CACAGGTATCTTGTCGTT
β-actin	Forward	CACGATGGAGGGGCCGGACTCATC
	Reverse	TAAAGACCTCTATGCCAACACAGT

PINK1 gene knocked out cells and mice by CRISPR/cas9

PINK1 (labeled by EGE-WL-008, Gene ID: 298575) is located on the reverse chain of chromosome 5, with a total length of 12.1kb. The gene contains two transcripts (1146 bp and 2020 bp respectively). In order to knock out the PINK1 gene, exons 2-8 with the length of about 7.5kb were selected to delete. Two sgRNA sequences were designed in the intron1 and the non-conserved sequence of 3'-UTR downstream. The PINK1 knockout mice were prepared by CRISPR/cas9 methods. The knockout strategy was shown in Figure 1.

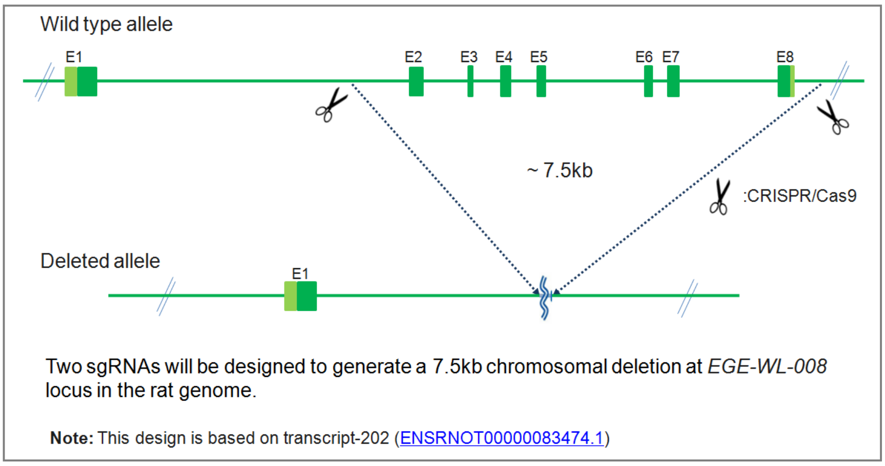

Figure 1. PINK1 gene knockout strategy

In order to ensure the efficiency of the designed cas9/sgRNA, the target site sequence was amplified by PCR and sequenced to ensure that the sgRNA recognition sequence is completely consistent with the target site sequence. The used PCR primers were shown in Table 2. Eight sgRNA sequences were designed according to the 5'- and 3'-domain sequence of target site sequence (Table 3). According to the sgRNA sequence, the corresponding primers were synthesized and connected into the pCS-3G vector by Gibson assembly methods. The vector products were transformed and sent to sequence for verifying the correctness. After detecting the activity of sgRNA, EGE-WL-008-sgRNA4 and EGE-WL-008-sgRNA9 (Figure 2) were used for the further experiments. Finally, the Cas9/sgRNA was microinjected into mouse fertilized eggs for knocking out the PINK1 gene.

Table 2. Primers used for validating the efficiency of the designed cas9/sgRNA

**Primer**	**Sequence (5'-3')**	**Product size**	**Tm (°C)**
EGE-WL-008-5'MSD-F	GCATCAAGCTTGGTACCGATCATG CAAGTGTTTTGGGTCCAC	984 (bp)	61
EGE-ML-008-5'MSD-R	ACTTAATCGTGGAGGATGATGCCT		62

	GAGGCCTTGTTTTAACTACC		
EGE-WL-008-3'MSD-F	GCATCAAGCTTGGTACCGATCACTT AAGCCTCTGGGGTGAGCATC	905 (bp)	65
EGE-ML-008-3'MSD-R	ACTTAATCGTGGAGGATGATTCAG CCTATCTCATCTCCCTGCACA		65

Table 3. The designed sgRNA sequences

**5'-Guide**	**sequence (5'-3')**	**3'-Guide**	**sequence(5'-3')**
Guide #1	CAGAGTAATCACCATCGACC AGG	Guide #9	GCGCTATTACTAAACGACTA AGG
Guide #2	GACCCATGCAGTTTACTACA TGG	Guide #10	ACACCATGACATCACAACGC AGG
Guide #3	ATCACTCACGGGGCCCAACG TGG	Guide #11	GGCCACTTACAAACTAAACC TGG
Guide #4	AGGTCIGTGTAACGCCACGT TGG	Guide #12	GCCCATCCATCTAAGT TCTA GGG
Guide #5	GACCTGAACAAATCCGTGTG AGG	Guide #13	TCTACCGCCTGAACTIGTTGA AGG
Guide #6	GAACCACCCTCTTATIGGGC TGG	Guide #14	GCACCTGCGTTGTGATGTCA TGG
Guide #7	CCCTAGTGACCACGGGGAAT GGG	Guide #15	AACCAGGTTTAGTTTGTAAG TGG
Guide #8	GCTGTCCCCTAGTGACCACG GGG	Guide #16	GTTCTGCCCTAGAACTTAGA TGG

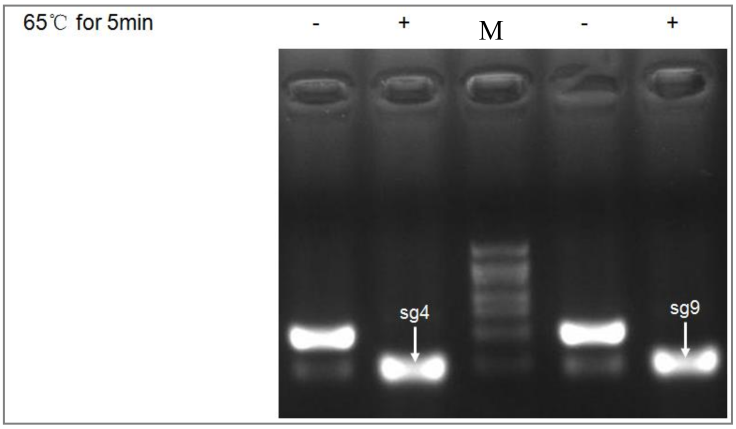

Figure 2. Conversion products of EGE-WL-008-sgRNA4 and EGE-WL-008-sgRNA9

M: marker, -: empty pCS-3G vector, +: pCS-3G vector with inserted sgRNA primers.
